# An Integrated Genomic and Expression Analysis of 7q Deletion in Splenic Marginal Zone Lymphoma

**DOI:** 10.1371/journal.pone.0044997

**Published:** 2012-09-13

**Authors:** A. James Watkins, Rifat A. Hamoudi, Naiyan Zeng, Qingguo Yan, Yuanxue Huang, Hongxiang Liu, Jianzhong Zhang, Esteban Braggio, Rafael Fonseca, Laurence de Leval, Peter G. Isaacson, Andrew Wotherspoon, Ellen D. McPhail, Ahmet Dogan, Ming-Qing Du

**Affiliations:** 1 Division of Molecular Histopathology, Department of Pathology, University of Cambridge, Cambridge, United Kingdom; 2 State Key Laboratory of Cancer Biology, Department of Pathology, Xijing Hospital, Xian, People’s Republic of China; 3 Department of Histopathology, Addenbrooke’s Hospital, Cambridge, United Kingdom; 4 Department of Pathology, 306 Hospital, Beijing, People’s Republic of China; 5 Comprehensive Cancer Centre, Mayo Clinic in Arizona, Scottsdale, Arizona, United States of America; 6 Division of Hematology, Cancer Center – Oncology, Mayo Clinic in Arizona, Scottsdale, Arizona, United States of America; 7 Institut Universitaire De Pathologie, Lausanne, Switzerland; 8 Department of Histopathology, University College London Hospital, London, United Kingdom; 9 Department of Histopathology, Royal Marsden Hospital, London, United Kingdom; 10 Department of Laboratory Medicine and Pathology, Mayo Clinic, Rochester, Minnesota, United States of America; University of Bonn, Institut of Experimental Hematology and Transfusion Medicine, Germany

## Abstract

Splenic marginal zone lymphoma (SMZL) is an indolent B-cell lymphoproliferative disorder characterised by 7q32 deletion, but the target genes of this deletion remain unknown. In order to elucidate the genetic target of this deletion, we performed an integrative analysis of the genetic, epigenetic, transcriptomic and miRNomic data. High resolution array comparative genomic hybridization of 56 cases of SMZL delineated a minimally deleted region (2.8 Mb) at 7q32, but showed no evidence of any cryptic homozygous deletion or recurrent breakpoint in this region. Integrated transcriptomic analysis confirmed significant under-expression of a number of genes in this region in cases of SMZL with deletion, several of which showed hypermethylation. In addition, a cluster of 8 miRNA in this region showed under-expression in cases with the deletion, and three (miR-182/96/183) were also significantly under-expressed (*P*<0.05) in SMZL relative to other lymphomas. Genomic sequencing of these miRNA and *IRF5*, a strong candidate gene, did not show any evidence of somatic mutation in SMZL. These observations provide valuable guidance for further characterisation of 7q deletion.

## Introduction

Splenic marginal zone lymphoma (SMZL) is a rare indolent B-cell non-Hodgkin’s lymphoma, being less than 2% of lymphoid malignancies [1], and the genetic basis underlying its development is poorly understood. SMZL lacks chromosome translocation that characterises other low grade B-cell lymphomas. Approximately, 30% of SMZL are characterised by heterozygous 7q deletion, which is also seen in splenic B-cell lymphoma/leukaemia unclassifiable but rarely in other lymphoma subtypes [2]–[4]. The genes targeted by this 7q deletion remain unknown. By BAC (bacterial artificial chromosome) array comparative genomic hybridisation (aCGH), we recently narrowed the minimally deleted region (MDR) to a 3.04 Mb region at 7q32 [4], which contains over 40 coding genes and a cluster of 6 microRNAs (miR-593, miR-129, the miR-182/96/183 polycistron and miR-335).

The 7q deletion may play an important role in the pathogenesis of SMZL. To investigate this, we searched for evidence of any potential tumour suppressor genes in the MDR. The classic tumour suppressor genes are often inactivated on both alleles by multiple mechanisms including homozygous deletion, heterozygous deletion and mutation, and transcriptional repression by promoter methylation. To ascertain the gene or genes targeted by the 7q deletion in SMZL, we sought evidence of homozygous deletion by gene resolution array CGH of chromosome 7. We also assessed the expression of miRNA and coding genes in the MDR and correlated their expression levels with the 7q deletion status. For the coding genes, we further correlated their expression with the methylation status. Finally, genomic sequencing was performed on the miRNA and key candidate genes in the MDR to search for inactivating mutation.

## Materials and Methods

### Case Selection

A total of 95 cases of SMZL were analysed in this study: 35 cases were the subject of a previous oligonucleotide aCGH study by the Mayo Clinic group [5] and the remaining 60 cases were the subject of BAC aCGH and interphase FISH (fluorescence in situ hybridisation) investigation by the Cambridge research group [4]. All were well characterised cases diagnosed on histological investigation of the splenic specimens by specialist haematopathologists according to the 2008 WHO classification of tumours haematopoietic and lymphoid tissues (Supplementary Table S1) [1]. The use of these archival tissues for research was approved by the local ethics committees. In each of the SMZL cases included in this study, patient had splenectomy and the genetic and epigenetic investigations described below were based on the splenic tissue specimens.

### Array Comparative Genomic Hybridization

A total of 56 case of SMZL were investigated by aCGH using oligonucleotide array platform in this study. A series of 35 SMZL (including 10 with 7q deletion) were examined using the Agilent aCGH 244A platform (6.4 Kb resolution) (Agilent Technologies, Palo Alto, California, USA) by the Mayo Clinic group in a previous study (http://www.ncbi.nlm.nih.gov/geo/, GSE35278) [5]. In addition, a customised oligonucleotide array was constructed (Agilent Technologies), with a 2 Kb resolution covering the whole chromosome 7q and further overlapping probes spanning all the miRNA at 7q32, including the miR-29a/29b1 polycistron. The miR-29a/29b1 polycistron lies distal to the MDR, but has been shown to be down-regulated in SMZL and other lymphomas [6], and was therefore also covered by overlapping probes. A further series of 21 SMZL (including 7 cases with 7q deletion) were investigated using this customised oligonucleotide array (GSE 21554) by the Cambridge group in this study.

The oligonucleotide array CGH was carried out essentially according to the manufacturer’s instructions. Briefly, genomic DNA was extracted from frozen tissues containing >60% tumour cells based on histological estimation with the assistance of CD20 and CD3 staining, together with retrospective review of the CGH profile. DNA (800 ng) was labelled with Cy5 (sample) and Cy3 (mixed normal reference) using the BioPrime® aCGH Labelling Kit (Invitrogen). Labelled genomic reactions were cleaned-up with purification columns (Invitrogen). The labelled DNA was mixed with Cot-1 DNA (Invitrogen), 10× Blocking Agent and 2× Hi-RPM Hybridization Buffer (Agilent) and hybridised to the array in a 60°C oven for 20 hours. Slides were scanned in an Agilent High Resolution-C scanner. Data was analysed using the Agilent Feature Extraction Software v10.5 and visualized in Genomic Workbench® Standard Edition (v.5.0.14).

Copy-number abnormalities (CNA) were calculated using the aberration detection module (ADM)-2 algorithm. Copy number variations (CNV) were identified and excluded from the analysis by reference to the database of genetic variation (Build GRCh37, Feb 2009: http://projects.tcag.ca).

### Expression Microarray Analysis

A total of 48 cases of SMZL (including 15 with 7q deletion) were analysed using the Affymetrix HG-U133 Plus 2.0 platform (Affymetrix, Santa Clara, California, USA). Arrays were performed according to the manufacturer’s instructions. Briefly, RNA was extracted from snap frozen tissues with >60% tumour cells using the RNeasy extraction kit (Qiagen) and subjected to DNAse treatment (Turbo DNAse kit, Ambion). RNA integrity was assessed using an Agilent 2100 Bioanalyzer. cDNA synthesis was carried out with 2 µg RNA using the GeneChip® One-Cycle cDNA Synthesis Kit (Affymetrix), followed by *in vitro* transcription with biotin-labelled nucleotides using GeneChip® IVT Labeling Kit. Biotinylated cRNA was purified and hybridized to the Affymetrix HG-U133 Plus 2.0 chips in a GeneChip® Hybridisation Oven 640 at 45°C for 14 hours. The arrays were then washed and stained using the Fluidics station 450 system (Affymetrix). The arrays were scanned using the Affymetrix GeneArray® Scanner 3000. Hybridisation and labelling controls were included according to the manufacturer’s instructions, and quality control analysis of microarrays was performed to published standards [7]–[9]. The data have been deposited with GEO (GSE21554, GSE35348).

### Bioinformatic Analysis of Expression Data

Raw gene expression data from Affymetrix.CEL files were uploaded to Bioconductor where a combined MAS5 and gcRMA normalization procedure was performed and used to filter out non-variant probes across all samples as described previously [9]. The gcRMA normalized data were imported into Genespring 7.3.1 and log-transformed. Expression levels of the genes on chromosome 7 were compared between cases of SMZL with and without 7q deletion by one-way ANOVA test with Benjamini Hochberg multiple testing correction. Genes with *P*<0.05 were considered to be differentially expressed. In addition, the differentially expressed genes were further filtered by fold changes to eliminate those with moderate changes <1.25×SD (standard deviation). In house software was written in R to plot the *P* value and fold change along the chromosome 7 sequence.

### Array Based Epigenetic Methylation Analysis

Epigenetic methylation analysis was performed using the Infinium® Human Methylation 27 array (Illumina, San Diego, California, USA). The array contained 27,568 CpG islands within the proximal promoter regions of transcription start sites of 14,475 RefSeq genes, including 12,883 well annotated genes (NCBI CCDS database: Build 36). The methylation array was carried out in 12 SMZL (6 cases with 7q deletion), 6 follicular lymphomas (FL) and 6 mantle cell lymphomas (MCL) as per the manufacturer’s instructions. Briefly, 2 µg genomic DNA extracted from frozen tissues with >60% tumour cells was bisulphite modified using the EZ DNA Methylation™ Kit (Zymo Research Corporation). Bisulphite modified DNA was then amplified using the MSM master mix (Illumina) and incubated at 37°C for 22 hours. Amplified DNA was then fragmented and hybridised to BeadChips in an Illumina Hybridisation Oven at 48°C for 18 hours. Following hybridisation, single base extension of hybridised DNA using hapten labelled bases was performed. Staining was then developed using immunochemical stains catalysed by the haptens, and the arrays washed. The chips were scanned using the BeadArray™ Reader (Illumina) and the BeadScan™ software (Illumina) using the Infinium Methylation Scan setting. The scanned data was then analysed in GenomeStudio™ (Illumina) using the Methylation analysis module. The data are available from GEO (GSE21554).

### Bioinformatic Analysis of Methylation Data

The scanned data was spatially normalised to correct for background noise using the Smethillium algorithm (http://bioinfo-out.curie.fr/projects/smethillium) within the R environment (www.r-project.org) [10]. Where multiple probes targeted different CpG loci relating to one gene, the probe predicted to have the greatest influence on gene expression (eg. at a transcriptional factor binding site, proximity to the promoters) was selected for analysis.

### Quantitative RT-PCR Analysis of miRNA

Total RNA was extracted from frozen tissues of 18 SMZL including 8 cases with 7q deletion, and 15 other low grade B-cell lymphomas including 7 FL, 4 MCL and 4 mucosa associated lymphoid tissue (MALT) lymphomas using the miRNEasy® kit (Qiagen). In each case, the frozen tissue used for RNA extraction contained at least 60% of tumour cells. The expression level of mature miR-593, miR-129, miR-182, miR-96, miR-183, miR-335, miR-29a, and miR-29b1 and their minor variants was investigated by qRT-PCR using the Qiagen miScript® system (Supplementary Table S2). Briefly, 1 µg of total RNA was reverse transcribed using the miScript® RT kit and 500 ng of the cDNA product was then subjected to quantitative PCR with the QuantiTect® SYBR Green system (Qiagen) using a BioRad IQ Light-Cycler. The PCR primers for the major and minor species of mature miR-593, miR-129, miR-182, miR-96, miR-183, miR-335, miR-29a, and miR-29b1 are detailed in Table S1. The reverse primer was a universal primer to a tag added during the RT step. In each case, the reaction was carried out in triplicate, and different runs were normalised against a common calibrator sample. The geometric mean of the expression of RNU6b and SCARNA17 was used as a reference and expression levels was calculated by the ΔΔCt method. Statistical analysis of expression between groups was performed using the Mann-Whitney test, and correlation of expression of major and minor species and of miRNA transcribed from the same polycistron using Pearson’s correlation co-efficient.

### Genomic Sequencing of miRNA and Coding Genes

Mutation analysis of miRNA was performed in 60 cases of SMZL. Primers flanking the predicted primary miRNA (pri-miRNA) (approximately 100 bp 5¢ and 3¢ of the predicted loop) were designed for miR-593, miR-129, miR-182, miR-96, miR-183, miR-335, miR-29a, and miR-29b1 (Table S3) according to the miRBase database (www.mirbase.org). PCR product was purified using EXO-Sap-IT (USB) and sequenced using the BigDye® Terminator v3.1 Cycle Sequencing kit on an ABI-3730 sequencer (Applied Biosystems). Sequences were aligned using SeqScape® v2.5 (Applied Biosystems). The nucleotide alterations identified were first excluded from known polymorphisms by searching the dbSNP database (Build 132) (www.ncbi.nlm.nih.gov/projects/SNP). Where indicated, PCR and sequencing of the DNA sample from the microdissected normal cells were carried out to ascertain whether the mutation was a somatic or germline change. The intact splenic capsule was manually microdissected under a light microscope as described previously [11] and used as a source of non-neoplastic DNA.

Similarly, *IRF5* was investigated for somatic mutation in SMZL by PCR and sequencing in 49 cases of SMZL, 28 FL, 8 MCL and 32 DLBCL (Table S4).

## Results

### High Resolution Array CGH Delineates a Common Region of Heterozygous Deletion at 7q32

**Figure 1:**
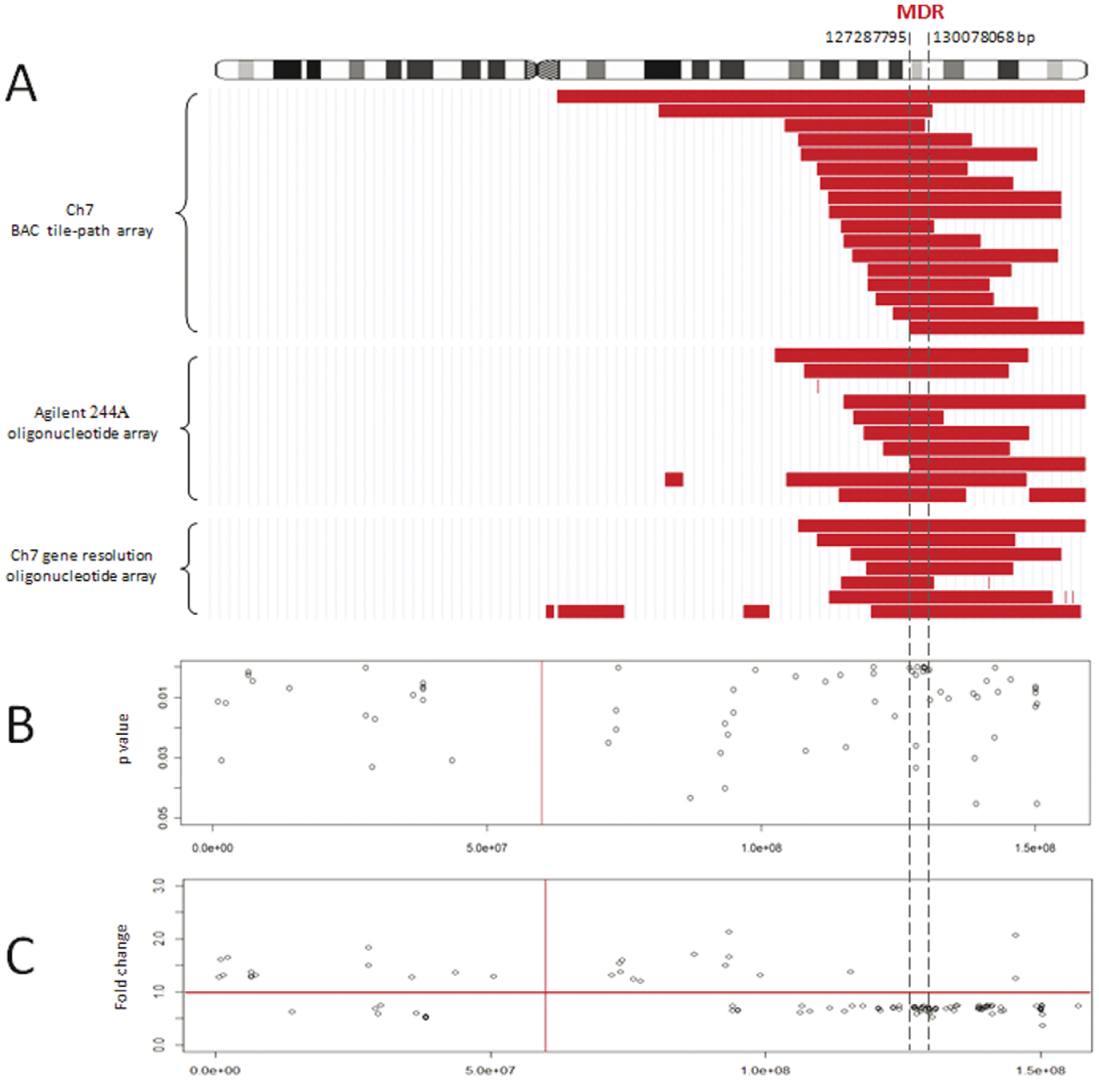
Investigation of 7q deletion in SMZL by array CGH and Affymetrix gene expression analyses. CGH using the chromosome 7 (Ch7) BAC tile-path array, Agilent 244A oligonucleotide array platform and chromosome 7 gene resolution oligonucleotide array maps the MDR to a 2.8 Mb region at 7q32 (Panel A). As shown by Affymetrix gene expression analyses, the genes on 7q, particularly those within the MDR are significantly under-expressed in SMZL with 7q deletion than those without the deletion. Panel B shows the genes significantly differentially expressed between SMZL with and without 7q deletion according to *P* values, while Panel C displays those with further fold change more than 1.25×SD (standard deviation).

Figure 1 summarised the 7q deletion in SMZL detected by CGH using a chromosome-7 BAC tile-path array (17 cases) and the Agilent aCGH 244A array CGH (10 cases) from our previous studies [4], [5]. There was no correlation between 7q deletion and patient’s survival (Supplementary Table S5). The combined analyses mapped the MDR to a region of 2.8 Mb (127,287795-130,078068 Mb, GRCh37), but showed no evidence of homozygous deletion within the MDR. To further investigate the possible existence of cryptic homozygous deletions that might have been under-detected by the above BAC tile-path and standard Agilent oligonucleotide array CGH, we investigated 21 cases of SMZL including 7 with 7q deletion using a customised oligonucleotide array with a 2 Kb resolution covering the whole chromosome 7q and gene resolution in the MDR. Despite this high resolution coverage, no evidence of cryptic or homozygous deletions of either miRNA or coding genes was observed in any of the cases investigated.

Within the MDR, there were 6 miRNA (miR-593, miR-129, miR-182, miR-96, miR-183, miR-335) with miR-29a/29b1 polycistron distal to the MDR, and 38 potential coding genes (Table 1).

**Table 1:** Summary of somatic mutation of the genes of the 7q MDR in human cancer.*

Gene	Full name	Mutation frequency		Nature of mutation	Cancer type
*SND1*	staphylococcal nuclease and tudor domain containing 1	4/93	4.3%	Missense,inframe insertion	Ovarian carcinoma’ primitive neuroestodermal tumour- medulloblastoma, laryngeal carcinoma
*LRRC4*	leucine rich repeat containing 4	4/154	2.6%	Missense,inframe deletion	Glioma, breast carcinoma, colon carcinoma, pancreatic carcinoma
*LEP*	leptin	0/90	0%	N/A	N/A
*RBM28*	RNA binding motif protein 28	2/92	2.2%	Missense,	Ovarian carcinoma
*IMPDH1*	IMP (inosine 5'-monophosphate) dehydrogenase 1	5/231	2.2%	Missense,	Skin melanoma, glioma, lung carcinoma,Ovarian carcinoma
*METTL2A*	methyltransferase like 2A	2/250	0.8%	Missense,	Mouth squamous carcinoma, pharyngeal carcinoma
*FAM71F1*	family with sequence similarity 71, member F1	2/70	2.8%	Missense, nonsense	Skin melanoma, mouth squamous carcinoma
*CCDC136*		2/25	8%	Missense,	Ovarian carcinoma
*FLNC*	filamin C, gamma	7/142	4.9%	Missense, nonsense	Skin melanoma, breast carcinoma, primitive neuroestodermal tumour-medulloblastoma, colon carcinoma, ovarian carcinoma
*KCP*	kielin/chordin-like protein	0/68	0%	N/A	N/A
*IRF5*	interferon regulatory factor 5	0/217	0%	N/A	N/A
*TNPO3*	transportin 3	2/219	0.9%	Missense, nonsense	Skin melanoma, lung carcinoma
*ATP6V1F*	ATPase, H+ transporting, lysosomal 14 kDa, V1 subunit F	1/91	1%	Missense,	Ovarian carcinoma
*TSPAN33*	tetraspanin 33	1/91	1%	Missense,	Ovarian carcinoma
*SMO*	smoothened, frizzled family receptor	32/2436	1.3%	Missense,Inframe insertion	Skin basal cell carcinoma, primitive neuroestodermal tumour- medulloblastoma, lung carcinoma, colon carcinoma, glioma, bile duct carcinoma
*AHCYL2*	adenosylhomocysteinase-like 2	3/71	4.2%	Missense, nonsense	laryngeal carcinoma, ovarian carcinoma
*NRF1*	nuclear respiratory factor 1	1/91	1%	Missense	Ovarian carcinoma
*UBE2H*	ubiquitin-conjugating enzyme E2H	1/217	0.5%	N/A	N/A
*ZC3HC1 (NIPA)*	zinc finger, C3HC-type containing 1	0/90	0%	N/A	N/A
*KLHDC10*	kelch domain containing 10	0/90	0%	N/A	N/A
*TMEM209*	transmembrane protein 209	2/92	2.2%	Missense,	Ovarian carcinoma, colon carcinoma
*CPA2*	carboxypeptidase A2 (pancreatic)	1/92	1%	Missense,	Mouth squamous carcinoma
*CPA4*	carboxypeptidase A4	3/517	0.6%	Missense, nonsense	laryngeal carcinoma, ovarian carcinoma
*CPA5*	carboxypeptidase A5	2/93	2.1%	Missense, nonsense	Ovarian carcinoma
*MEST*	mesoderm specific transcript homolog	2/516	O.6%	Missense, nonsense	Ovarian carcinoma
*TSGA13*	testis specific, 13	3/93	3.2%	Missense,	laryngeal carcinoma, ovarian carcinoma
*KLF14*	Kruppel-like factor 14	1/91	1%	Missense,	Mouth squamous carcinoma

* From the COSMIC database (http://www.sanger.ac.uk/genetics/CGP/cosmic/).

### Under-expression of miRNA of the MDR in SMZL with 7q Deletion

**Figure 2:**
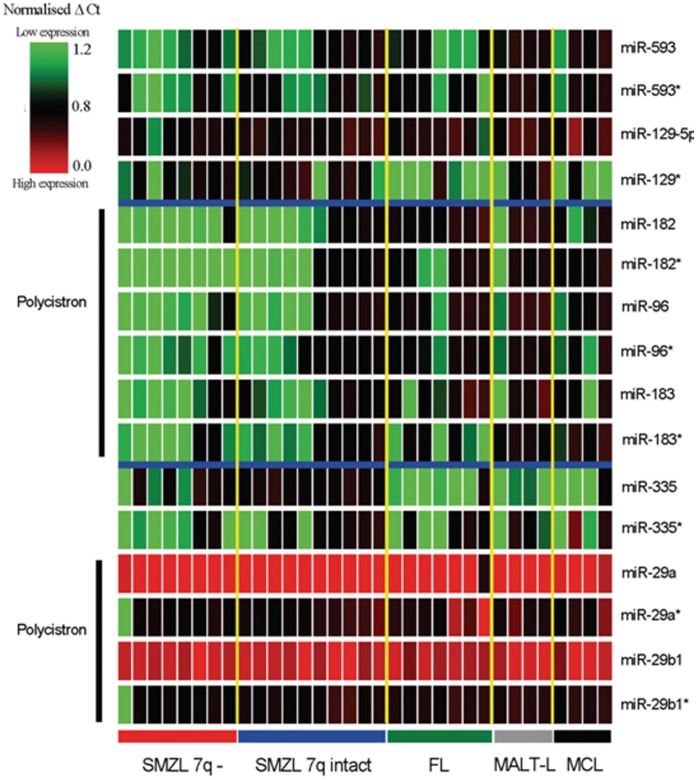
Heatmap illustration of the qRT-PCR of the 6 miRNA (miR-593, miR-129, miR-182, miR-96, miR-183, miR-335) within the MDR and 2 miRNA (miR-29a/29b1 polycistron) distal to the MDR. The results show that the expression of these mature miRNAs is in general lower in SMZL with 7q deletion than those without the deletion, although not statistically significant with the exception of miR-29a. *minor miRNA species, FL: follicular lymphoma; MCL: mantle cell lymphoma; MALT-L: MALT lymphoma.

We first investigated whether the expression of the miRNA within the 7q32 MDR was impaired by 7q deletion by comparing their expression between SMZL with (n = 8) and without 7q deletion (n = 10) using qRT-PCR. In general, 1) there was a significant correlation between the expression of major and minor species of the miRNA (*P*<0.001) and also among the miRNA transcribed from the same polycistron (p<0.001), indicating good quality of experimental data; and 2) the expression of mature miR-593, miR-129, miR-182, miR-96, miR-183, miR-335, miR-29a and miR-29b1 was consistently reduced in cases with 7q deletion as compared with those without the deletion (Figure 2 & Figure S1), albeit not statistically significant with the exception of miR-29a, most likely due to small number of cases investigated.

It is thus likely that 7q deletion underlies the reduced expression of these miRNA. Interestingly, the expression of these miRNA was also different among SMZL, FL, MCL and MALT lymphoma entities (Figure 2), with SMZL in general showing much lower expression levels. In particular, miRNA in the miR-182/miR-96/miR-183 polycistron showed statistically significant under-expression in SMZL (Figure S1, *P*<0.05).

### Genomic Sequencing of miRNA of the MDR Revealed No Evidence of Somatic Mutation

To further search for evidence that miRNA may be the target of 7q deletion in SMZL, we performed PCR and sequenced the miR-593, miR-129, miR-182, miR-96, miR-183, miR-335, miR-29a and miR-29b1 genes in 23 cases of SMZL. This initial screening revealed a SNP, rs76481776 (G to A substitution at nucleotide position 106), in the primary miR-182 in 5 cases of SMZL but showed no nucleotide alterations in any of the other primary miRNA investigated. In addition, two novel SNPs were observed in the 3¢ region distal to the pri-miR-182 hairpin. Subsequent screening showed the rs76481776 (G106A) variant in 12 (20%) of the 60 SMZL investigated. There was no association between the rs76481776 substitution and 7q deletion, or immunoglobulin heavy chain somatic mutation status. The rs76481776 substitution was also seen frequently in FL (20%), HCL (13%), MALT lymphoma (13%), PTCL-NOS (9%), less frequently in CLL (5%), DLBCL (4%) and MCL (3%), but not in lymphoplasmacytic lymphoma and 3 lymphoma cell lines (Figure S2).

### Under-expression of Coding Genes at 7q32 in SMZL with 7q Deletion

To investigate whether any of the coding genes at 7q32 may be the target of 7q deletion, we compared the expression of the genes on chromosome 7 between SMZL with (n = 15) and without 7q deletion (n = 33) by analysis of the Affymetrix gene expression profile. In general, the genes on 7q, particularly those within the MDR, were under-expressed in cases with 7q deletion, in keeping with the relatively large and heterogeneous regions affected by the deletion (Figure 1). Within the MDR, there were 38 potential coding genes, and 37 of them were represented on the U133 Plus 2.0 array (*PRRT4* was not represented). Of these 37 genes, 18 (*TNPO3*, *KLHDC10, RBM28, NIPA, SND1, TSGA14, TMEM209, UBE2H, NRF1, NAG8, ATP6V1F, CALU, AHCYL2, COPG2, HIG2, TSPAN33, IMPDH1* and *IRF5*) were significantly under-expressed in SMZL with 7q deletion in comparison with those without the deletion (Figure S3, Supplementary Table S6) (*P*<0.05). In 9 (24%) of these genes, the fold changes were more than >1.25×standard deviation. As a baseline control, only 7 (11.7%) of the 60 genes on 7p were expressed at levels significantly lower in cases with 7q deletion than those without the deletion according to the same criteria.

### Integrative Epigenetic and Transcriptomic Analysis of the Genes within MDR

Transcriptional repression by promoter methylation is one of the common mechanisms underlying the inactivation of tumour suppressor genes. To search for potential evidence of gene inactivation by promoter methylation, we correlated the levels of promoter methylation and expression of the genes within the MDR in SMZL. Among the 37 genes which were represented in both the methylation and Affymetrix expression arrays, *CPA4, OPN1SW, NAG8, LRRC4, CPA5, CPA2, TSGA13, CPA1, C7orf45* and *NYD-SP18* consistently showed high levels of methylation but low gene expression in each of the SMZL with 7 q deletion (Figure S4). To examine whether this was specific to SMZL, we investigated FL and MCL, and correlations between high levels of methylation and low expression of these genes were also found in these lymphoma entities.

### Sequencing of IRF5 in SMZL Reveals Recurrent Polymorphism but No Evidence of Somatic Mutation

Among the coding genes within the MDR, *IRF5*, an essential transcriptional factor activated by TLR signalling, is critical for antiviral immunity and functions as a tumour suppressor [12]. In view of the putative role of viral infection in pathogenesis of SMZL and recent finding of activating mutations in *MYD88* (an adaptor in TLR signalling) in SMZL, we investigated whether *IRF5* was targeted by mutation. By sequencing the coding exons (2–9) of *IRF5*, we identified an in-frame deletion polymorphism in exon 6 (rs60344245) in 29/49 (59%) of SMZL investigated, with heterozygous mutation in 14 (28.6%) and homozygous mutation in 15 (30.6%) (Figure S5). The polymorphism was similarly seen in FL (18/28 = 64%, heterozygous deletion in 14/28 = 50% and homozygous deletion in 4/28 = 14.2%), MCL (6/8 = 75%, heterozygous deletion in 3/8 = 37.5% and homozygous deletion in 3/8 = 37.5%) and diffuse large B-cell lymphoma (22/32 = 68%, heterozygous deletion in 15/32 = 46.8% and homozygous deletion in 7/32 = 21.9%) (Figure S5). The frequencies of the IRF5 polymorphism in these lymphomas were higher or similar to those (49%) described in healthy German blood donors [13].

## Discussion

### The Characteristics of 7q Deletion in SMZL

By extensive high-resolution array CGH analyses of a large cohort of SMZL, the present study mapped the MDR of 7q deletion in SMZL to a region of 2.8 Mb (127,287795-130,078068 Mb, GRCh37). There are several features associated with the deletion, which are worth pointing out in considering the candidate genes targeted by the deletion. First, there was no evidence of recurrent breakpoints or cryptic deletion, which might indicate a particular single gene as the target of the deletion. This finding raises the probability that multiple genes are targeted by the deletion. Second, there was no evidence of homozygous deletion at 7q32 in SMZL. Thus, it is unknown whether the gene targeted by 7q deletion is a classic tumour suppressor gene and required to be inactivated on both alleles, and if so, an alternative mechanism of inactivation must be responsible. Finally, the SMZL with 7q deletion do not constitute a distinct subgroup as shown by integrated clinicopathological and gene expression microarray analyses [4], [14]–[16]. This suggests the possibility of presence of common molecular mechanisms between SMZL with and without 7q deletion. It is possible that the gene targeted by 7q deletion is inactivated by somatic mutation and/or promoter methylation in SMZL lacking the deletion. Alternatively, the molecular mechanism/cellular pathway deregulated by the 7q deletion may be similarly altered by other genetic events in cases without the deletion. Lastly, it is also possible that the genes targeted by the 7q deletion do not play a predominant role in the pathogenesis of SMZL, thus not bearing a major impact on gene expression profiles and clinicopathological presentation.

### miRNA as Possible Candidate Targets of 7q Deletion in SMZL

As shown by qRT-PCR, there was a clear trend of under-expression of all the miRNA within the MDR in SMZL with 7q deletion as compared with those without the deletion. In addition, the expression of miR-29a, which was located distal to the MDR, was significantly reduced in SMZL with 7q deletion than those without the deletion. Under-expression of miR-29a in SMZL has also been reported in previous studies [6], [17]. Although miR-29 was outside of the MDR, the region of 7q deletion was considerably heterogeneous and in 33 of the 34 cases of SMZL with 7q deletion investigated in this study, miR-29 was within the deleted region. It is thus likely that 7q deletion underlies the reduced expression of all these miRNAs. Interestingly, the expression of these miRNA, particularly miR-183/96/182 (which are transcribed from the same polycistron, *P*<0.05) was significant lower in SMZL than FL, MCL and MALT lymphoma. A reduced expression of miR-182/96/183 was also noted in cases of SMZL lacking the 7q32 deletion (Figure S1), suggesting presence of other molecular mechanisms that down-regulate the expression of these miRNAs. Together, these findings suggest an association of these miRNA expression changes with SMZL.

The normal cell counterpart of SMZL is unknown, so it is not possible to directly compare miRNA expression between SMZL and the cell of its origin. Several studies have investigated the miRNome of normal B-cell subsets by expression microarrays and shown variable expression of miR-182/96/183 among different B-cell subsets, being expressed highly in the germinal centre B-cells, but moderately in the naïve and memory B-cells [18], [19] suggesting an important role of these miRNA in B-cell biology. The findings of reduced expression of these miRNA in SMZL relative to other lymphoma subtypes arising from various stages of B-cell maturation suggest that these changes were lymphoma associated events rather than reflection of differential expression patterns between the normal cell counterparts of these lymphomas. By comparing with non-neoplastic splenic marginal zone B-cells, Peveling-Oberhag et al also identified a number of miRNAs showing significantly altered expression in SMZL, further implicating a role of miRNA deregulation in the lymphoma pathogenesis [20].

Alteration in miRNA sequences is another mechanism underlying miRNA mediated oncogenesis [21]. We carried out sequence analyses of all the 6 miRNA in the MDR together with miR-29a/29b1 polycistron that lies distal to the MDR in 23 SMZL, but found no evidence of somatic mutation. A single nucleotide polymorphism (rs76481776, G106A) was seen in the primary miR-182 sequence. The polymorphism was functional since it affected the expression of miR-182 as well as its target genes [22]. In line with this, analysis of the predicted secondary structure of the miR-182 G106A variant using RNA-Fold (www.rna.tbi.univie.ac.at/cgi-bin/RNAfold.cgi) reveals that the G→A substitution replaces a G:U wobble base pair with a stronger A:U pair in the double strand stem and this also impacts on the neighbouring base pairs close to the terminal overhangs (Figure S3). This region of pri-miRNA is critical for DGCR8 binding and subsequent accurate Drosha cleavage [23]. The substitution may affect the pri-miR-182 processing, thus the level of its expression. It is interesting to note that the miR-182 G106A polymorphism was at variable frequencies among different lymphoma subtypes investigated, and the frequencies of the polymorphism in SMZL (20%) and FL (20%) were much higher than that (12.7%) in a Spanish control population (n = 341) studied so far [22], but the difference was not statistical significant. Nonetheless, it remains to be investigated whether any of the miRNA at 7q32 is the target of 7q deletion. This is particularly enigmatic since the miR-182/96/183 polycistron were frequently over-expressed and thought to act as oncogenes in solid tumours [24]–[27]. However, the biological effect of miRNA expression is cell type and context dependent, and miR-182 has also been shown to act as a tumour suppressor in lung cancer [28], and it remains a possibility that these miRNA may be important in the pathogenesis of SMZL.

### Coding Genes as Possible Candidate Targets of 7q Deletion in SMZL

Among of the 37 coding genes within the MDR, 18 were significantly under-expressed in SMZL with the 7q deletion in comparison with those without the deletion. In 10 of these genes, their reduced expression appeared to be associated with epigenetic methylation as shown by integrated analyses of the gene expression and methylation microarray data, suggesting that promoter methylation may underpin their reduced expression. However, similar correlations were also seen in FL and MCL. It remains to be investigated whether these correlations are associated with lymphoma development or a common feature of their normal cell counterparts. In view of lack of clear evidence that pinpoints the genes likely targeted by the deletion, we further searched for somatic mutations in a favourite candidate gene *IRF5*, encoding a transcriptional factor activated by the TLR signalling. IRF5 is critical for antiviral immunity and functions as a tumour suppresser [12]. However, sequencing of all the coding exons of *IRF5* in 49 cases of SMZL showed no evidence of somatic mutation. The deletion polymorphism in *IRF5* exon 6 (rs60344245) found in SMZL was also seen at comparable frequencies in other B-cell lymphomas and a normal control population from Germany [13]. There are a number of SNPs in the *IRF5* gene including several known functional SNPs in both the transcriptional regulatory and coding regions. Several haplotypes of these *IRF5* variants were associated with distinct risks to human lupus and Wegener’s granulomatosis [13], [29]. It remains to be investigated whether any of the IRF5 haplotypes or SNPs is associated with lymphoma development.

To help the identification of the target genes of 7q deletion in SMZL, we searched the somatic mutation data in the COSMIC (Catalogue of Somatic Mutations in Cancer) database (http://www.sanger.ac.uk/genetics/CGP/cosmic/). The majority of the coding genes within the 7q MDR in SMZL are somatically mutated in human cancer although their incidence is relatively low and the functional impact of these mutations is unknown (Table 1). The somatic mutations in *FAM71F1*, *FLNC*, *TNP03*, *AHCYL2*, *CPA4* and *MEST* involves both missense and nonsense changes, suggesting that these genes might be targeted for inactivation. Of these genes, *FLNC* is particularly interesting since it is frequently repressed at the transcriptional level by promoter methylation in several human cancer types [30]–[33]. It remains to be investigated whether *FLNC*, and/or any of the other genes in the 7q MDR are the target of the deletion in SMZL. In view of the wide availability of massive parallel sequencing, the time for this discovery will not be too far in the near future.

In summary, our integrated analyses of 7q deletion by genomic, expression and methylation profiling showed that the expression of several genes within the MDR was significantly affected by the deletion. Our sequencing analyses also excluded the possibility of several potential candidate genes as the target of 7q deletion. The data presented in this study provide valuable guidance for further characterisation of 7q deletion.

## Supporting Information

Figure S1 **Comparison of miRNA expression between SMZL, FL, MCL and MALT lymphoma by qRT-PCR.** miR-593, miR-129, miR-182, miR-96, miR-183, miR-335 are within the MDR defined in this study, while miR-29a/29b1 polycistron is distal to the MDR. In general, the expression of these mature miRNAs is lower in SMZL with 7q deletion than those without the deletion, although not statistically significant with the exception of miR-29a. In addition, the express of these miRNA is also lower in SMZL than FL, MCL and MALT lymphoma. SMZL = Splenic marginal zone lymphoma; 7q− = 7q32 deletion; 7q+ = 7q32 intact; FL = Follicular lymphoma, MCL = Mantle cell lymphoma, MALT = MALT lymphoma.
(TIF)

Figure S2 **A functional polymorphism (G106A substitution) in pri-miR-182.** A: Sequencing shows presence of G106A substitution in both SMZL and microdissected normal cells (the intact splenic capsule); B: RNAFold analysis of the G106A polymorphism shows stronger stem structure at the site of the mutation. Coloured scale displays the predicted base pairing probability; C: The predicted hairpin loop of pri-miR-182 showing the site of the polymorphism; D: Frequency of the polymorphism in SMZL and other lymphomas. SMZL: splenic marginal zone lymphoma; FL: follicular lymphoma; MCL: mantle cell lymphoma; CLL: chronic lymphocytic leukaemia/lymphoma; MALT: MALT lymphoma; TCL: peripheral T-cell lymphoma not otherwise specified (PTCL-NOS); DLBCL: diffuse large B-cell lymphoma; HCL: hairy cell leukaemia; LPL: lymphoplasmacytic lymphoma.
(TIF)

Figure S3 **Comparison of the expression of coding genes in MDR between 7q deletion positive and negative SMZL.** Of these 38 genes within the MDR, 37 are represented on the Affymetrix U133 Plus 2.0 array, and 18 of these genes (*TNPO3*, *KLHDC10, RBM28, NIPA, SND1, TSGA14, TMEM209, UBE2H, NRF1, NAG8, ATP6V1F, CALU, AHCYL2, COPG2, HIG2, TSPAN33, IMPDH1* and *IRF5*) are significantly under-expressed in SMZL with 7q deletion in comparison with those without the deletion (*P*<0.05).
(TIF)

Figure S4 **Correlation of the expression and methylation levels of the genes within MDR in SMZL.** Among the 37 genes which were represented in both the methylation and Affymetrix expression arrays, *CPA4, OPN1SW, NAG8, LRRC4, CPA5, CPA2, TSGA13, CPA1, C7orf45* and *NYD-SP18* were consistently showed high levels of methylation but low gene expression in each of the SMZL with 7 q deletion. The data shown in this figure are the mean of normalised expression and methylation values.
(TIF)

Figure S5 **The IRF5 exon 6 in-frame 30 bp deletion polymorphism (rs60344245) in SMZL.** Panel A shows examples of the SNP, panel B displays the frequency of the SNP in SMZL, FL, MCL and DBLCL. There is no significant difference in the frequency of the polymorphism (rs60344245) among these lymphoma subtypes. SMZL: splenic marginal zone lymphoma; FL: follicular lymphoma; MCL: mantle cell lymphoma; DLBCL: diffuse large B-cell lymphoma.
(TIF)

Table S1 **Summary of SMZL used for CGH, gene expression and epigenetic methylation microarray analyses.**
(XLS)

Table S2 **Primers used for quantitative RT-PCR of miRNAs.**
(DOC)

Table S3 **Primers used for genomic sequence analysis of miRNA.**
(DOC)

Table S4 **Primers used for sequence analysis of** ***IRF5*** **.**
(DOC)

Table S5 **Univariate analysis for prognosis by Kaplan-Meier method.**
(DOC)
