# Supplementary figures and images for: An Integrated Genomic and Expression Analysis of 7q Deletion in Splenic Marginal Zone Lymphoma
Source: PLoS One. 2012 Sep 13;7(9):e44997. doi: 10.1371/journal.pone.0044997 (PMC3441634; doi:10.1371/journal.pone.0044997)

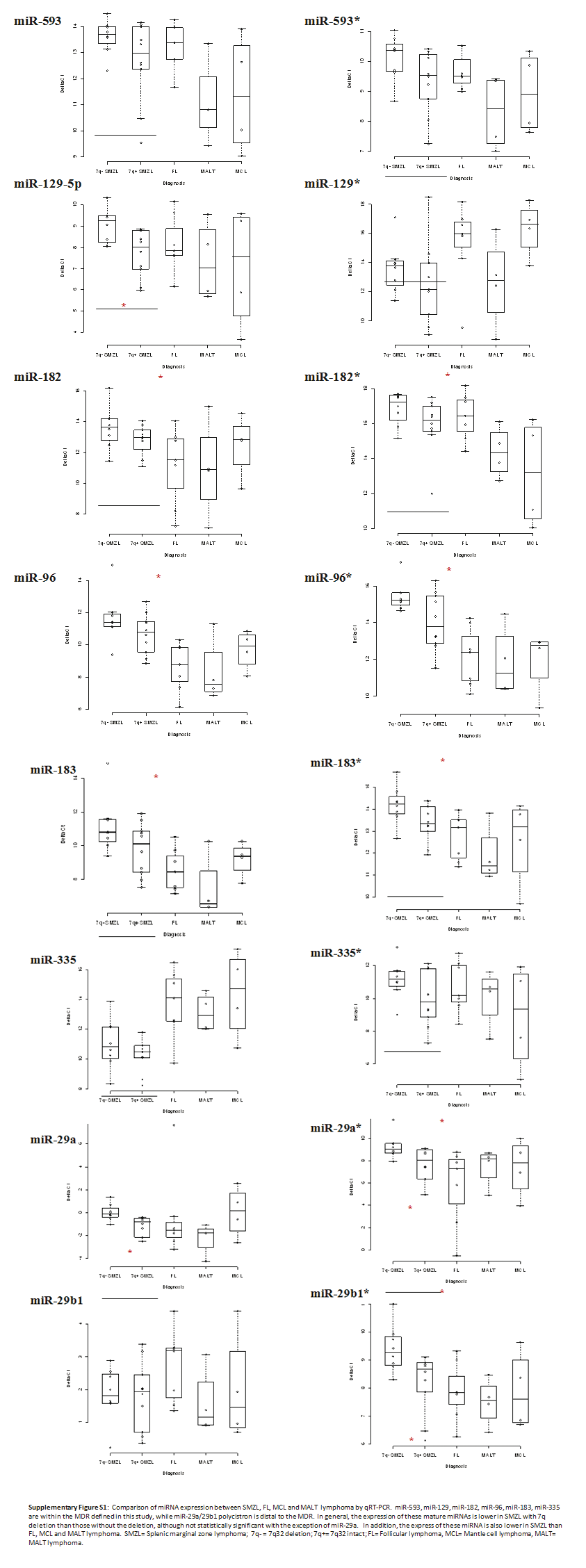

Supplement: Figure S1 — Comparison of miRNA expression between SMZL, FL, MCL and MALT lymphoma by qRT-PCR. miR-593, miR-129, miR-182, miR-96, miR-183, miR-335 are within the MDR defined in this study, while miR-29a/29b1 polycistron is distal to the MDR. In general, the expression of these mature miRNAs is lower in SMZL with 7q deletion than those without the deletion, although not statistically significant with the exception of miR-29a. In addition, the express of these miRNA is also lower in SMZL than FL, MCL and MALT lymphoma. SMZL = Splenic marginal zone lymphoma; 7q− = 7q32 deletion; 7q+ = 7q32 intact; FL = Follicular lymphoma, MCL = Mantle cell lymphoma, MALT = MALT lymphoma. (TIF) [file pone.0044997.s001.tif]

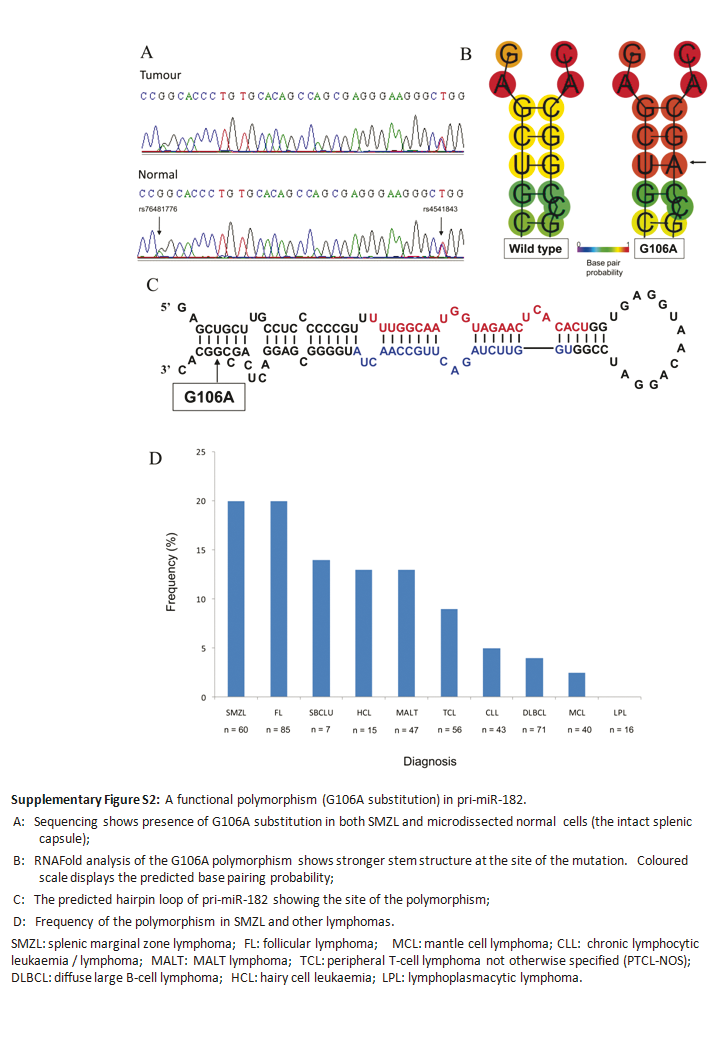

Supplement: Figure S2 — A functional polymorphism (G106A substitution) in pri-miR-182. A: Sequencing shows presence of G106A substitution in both SMZL and microdissected normal cells (the intact splenic capsule); B: RNAFold analysis of the G106A polymorphism shows stronger stem structure at the site of the mutation. Coloured scale displays the predicted base pairing probability; C: The predicted hairpin loop of pri-miR-182 showing the site of the polymorphism; D: Frequency of the polymorphism in SMZL and other lymphomas. SMZL: splenic marginal zone lymphoma; FL: follicular lymphoma; MCL: mantle cell lymphoma; CLL: chronic lymphocytic leukaemia/lymphoma; MALT: MALT lymphoma; TCL: peripheral T-cell lymphoma not otherwise specified (PTCL-NOS); DLBCL: diffuse large B-cell lymphoma; HCL: hairy cell leukaemia; LPL: lymphoplasmacytic lymphoma. (TIF) [file pone.0044997.s002.tif]

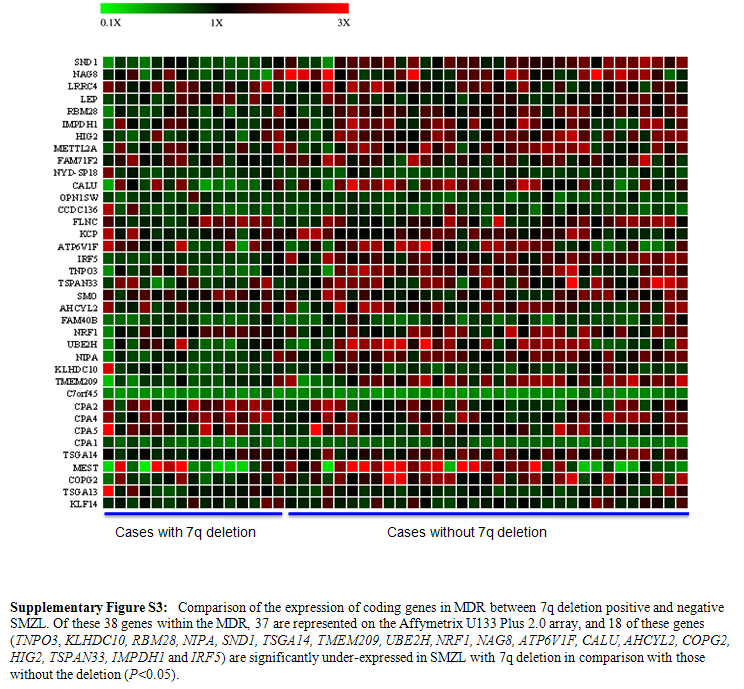

Supplement: Figure S3 — Comparison of the expression of coding genes in MDR between 7q deletion positive and negative SMZL. Of these 38 genes within the MDR, 37 are represented on the Affymetrix U133 Plus 2.0 array, and 18 of these genes (TNPO3, KLHDC10, RBM28, NIPA, SND1, TSGA14, TMEM209, UBE2H, NRF1, NAG8, ATP6V1F, CALU, AHCYL2, COPG2, HIG2, TSPAN33, IMPDH1 and IRF5) are significantly under-expressed in SMZL with 7q deletion in comparison with those without the deletion (P<0.05). (TIF) [file pone.0044997.s003.tif]

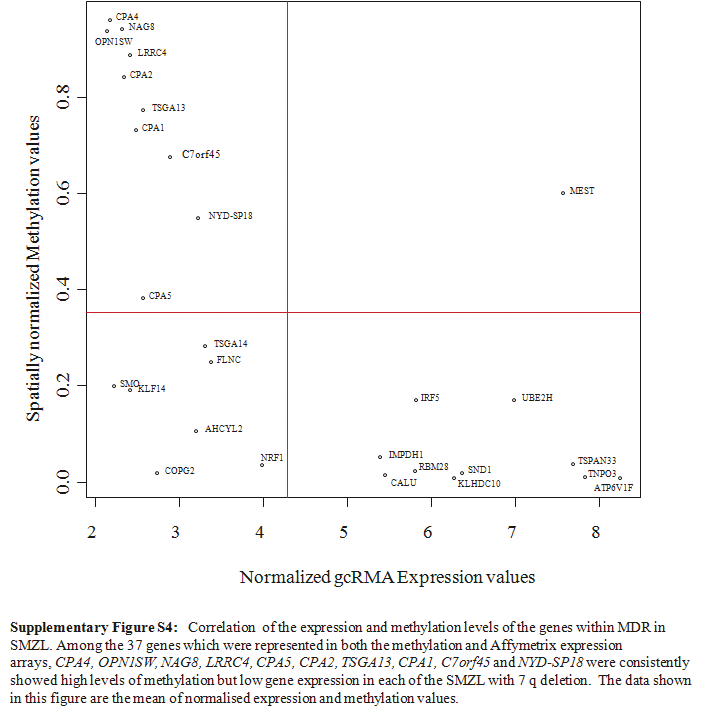

Supplement: Figure S4 — Correlation of the expression and methylation levels of the genes within MDR in SMZL. Among the 37 genes which were represented in both the methylation and Affymetrix expression arrays, CPA4, OPN1SW, NAG8, LRRC4, CPA5, CPA2, TSGA13, CPA1, C7orf45 and NYD-SP18 were consistently showed high levels of methylation but low gene expression in each of the SMZL with 7 q deletion. The data shown in this figure are the mean of normalised expression and methylation values. (TIF) [file pone.0044997.s004.tif]

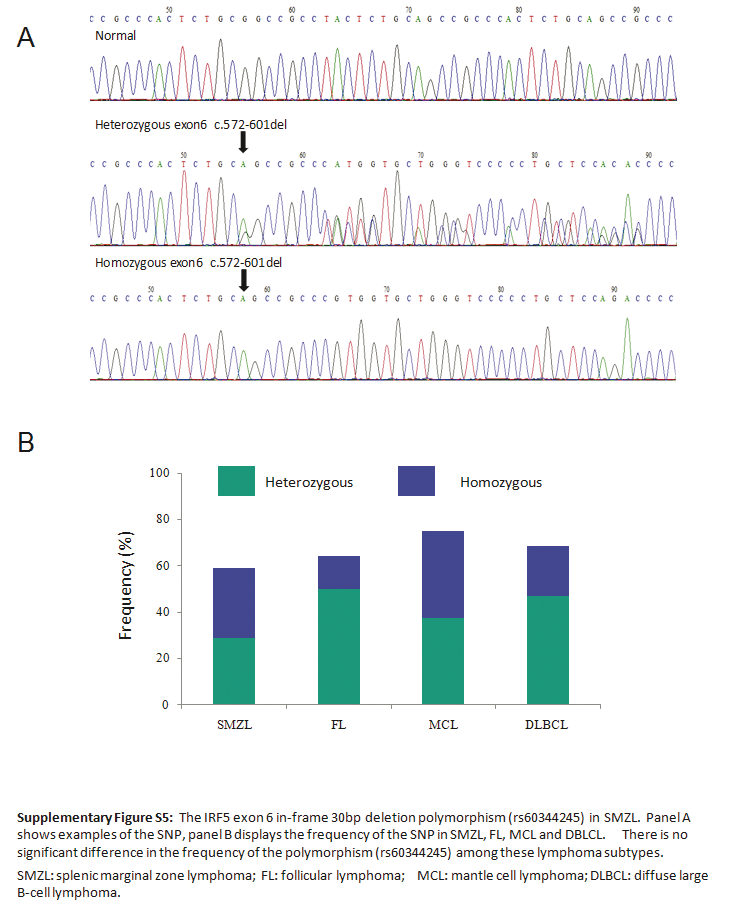

Supplement: Figure S5 — The IRF5 exon 6 in-frame 30 bp deletion polymorphism (rs60344245) in SMZL. Panel A shows examples of the SNP, panel B displays the frequency of the SNP in SMZL, FL, MCL and DBLCL. There is no significant difference in the frequency of the polymorphism (rs60344245) among these lymphoma subtypes. SMZL: splenic marginal zone lymphoma; FL: follicular lymphoma; MCL: mantle cell lymphoma; DLBCL: diffuse large B-cell lymphoma. (TIF) [file pone.0044997.s005.tif]
