# Supplementary material for: An Integrated Genomic and Expression Analysis of 7q Deletion in Splenic Marginal Zone Lymphoma
Source: PLoS One. 2012 Sep 13;7(9):e44997. doi: 10.1371/journal.pone.0044997 (PMC3441634; doi:10.1371/journal.pone.0044997)
Supplement: Table S2 — Primers used for quantitative RT-PCR of miRNAs. (DOC) [file pone.0044997.s007.doc]

**Supplementary Table S2**: Primers used for quantitative RT-PCR of miRNAs

| **miRNA** | **Sequence** | **PCR Conditions** |
| --- | --- | --- |
| Hsa-miR-593 | UGUCUCUGCUGGGGUUUCU | **Step 1**: 1 Cycle  15mins at 95°C  **Step 2**: x40 Cycles  15s at 94°C  30s at 55°C  30s at 70°C (Acquisition step)  **Step 3**: Melt Curve analysis (x 80 cycles)  55-95 °C in 0.5°C increments per cycle |
| Hsa-miR-593* | AGGCACCAGCCAGGCAUUGCUCAGC |
| Hsa-miR-129_5p | CUUUUUGCGGUCUGGGCUUGC |
| Hsa-miR-129* | AAGCCCUUACCCCAAAAAGUAU |
| Hsa-miR-182 | UUUGGCAAUGGUAGAACUCACACU |
| Hsa-miR-182* | UGGUUCUAGACUUGCCAACUA |
| Hsa-miR-96 | UUUGGCACUAGCACAUUUUUGCU |
| Hsa-miR-96* | AAUCAUGUGCAGUGCCAAUAUG |
| Hsa-miR-183 | UAUGGCACUGGUAGAAUUCACU |
| Hsa-miR-183* | GUGAAUUACCGAAGGGCCAUAA |
| Hsa-miR-335 | UCAAGAGCAAUAACGAAAAAUGU |
| Hsa-miR-335* | UUUUUCAUUAUUGCUCCUGACC |
| Hsa-miR-29a | UAGCACCAUCUGAAAUCGGUUA |
| Hsa-miR-29a* | ACUGAUUUCUUUUGGUGUUCAG |
| Hsa-miR-29b1 | UAGCACCAUUUGAAAUCAGUGUU |
| Hsa-miR-29b1* | GCUGGUUUCAUAUGGUGGUUUAGA |
