# Supplementary material for: An Integrated Genomic and Expression Analysis of 7q Deletion in Splenic Marginal Zone Lymphoma
Source: PLoS One. 2012 Sep 13;7(9):e44997. doi: 10.1371/journal.pone.0044997 (PMC3441634; doi:10.1371/journal.pone.0044997)
Supplement: Table S3 — Primers used for genomic sequence analysis of miRNA. (DOC) [file pone.0044997.s008.doc]

**Supplementary Table S3**: Primers used for genomic sequence analysis of miRNA.

| **miRNA** | **Primer sequence** | **Amplicon size (bp)** | **PCR Conditions** |
| --- | --- | --- | --- |
| Hsa-miR-593- F | 5’TGGGTGGAGCTCTGAGTCTCTT3’ | 406 | **Step 1**: 1 cycle  10mins at 95°C  **Step 2**: x10 cycles (Touchdown- 1°C /cycle-65-55°C)  1min at 95°C  1min at 65°C  1.30min at 72°C  **Step 3**: x25 cycles  1min at 95°C  1min at 55°C  1.30min at 72°C  **Step 4**: 1 cycle  10mins at 72°C |
| Hsa-miR-593- R | 5’GTAAGCGGTTGGAGGTGTAGTC3’ |
| Hsa-miR-129_1- F | 5’GCCATGGGATGGCTGCTGTCTC3’ | 153 |
| Hsa-miR-129_1- R | 5’CTGTGACTCACCTGGAGCCCTT3’ |
| Hsa-miR-182- F | 5’CCTGGACCATCCTAACTGTCTC3’ | 447 |
| Hsa-miR-182- R | 5’CTCACTCCTCGATTCAGACTCC3’ |
| Hsa-miR-182- F† | 5’GCAATGGTAGAACTCACACTGG3’ | 161 |
| Hsa-miR-182- R† | 5’GACCTGAGTCCCCTCCTTCCTC3’ |
| Hsa-miR-96- F | 5’GAATTACCGAAGGGCCATAAAC3’ | 541 |
| Hsa-miR-96- R | 5’CTTTCCAACCCACGGCACCATT3’ |
| Hsa-miR-183- F | 5’GTCTCCTTGAAGGTCATCTTGG3’ | 464 |
| Hsa-miR-183- R | 5’ACAAGCAAAAATGTGCTAGTGC3’ |
| Hsa-miR-335- F | 5’TCCTTACCATCCCTGATTTCAT3’ | 400 |
| Hsa-miR-335- R | 5’CTGGAAGTAACCCAAGCCAACA3’ |
| Hsa-miR-29a- F | 5’TCAAGGATACCAAGGGATGAAT3’ | 325 |
| Hsa-miR-29a- R | 5’ACGGTCACCAATACATTTCCTC3’ |
| Hsa-miR-29b1- F | 5’CATGCTCTCCCATCAATAACAA3’ | 315 |
| Hsa-miR-29b1- R | 5’TACTTCAGAGCTGTCCCATTCA3’ |

† = This primer pairs yields a smaller amplicon and was used in cases where only formalin fixed paraffin embedded tissues were available.

F: forward, R: Reverse.
