# Supplementary material for: An Integrated Genomic and Expression Analysis of 7q Deletion in Splenic Marginal Zone Lymphoma
Source: PLoS One. 2012 Sep 13;7(9):e44997. doi: 10.1371/journal.pone.0044997 (PMC3441634; doi:10.1371/journal.pone.0044997)
Supplement: Table S4 — Primers used for sequence analysis of IRF5 . (DOC) [file pone.0044997.s009.doc]

**Supplementary Table S4**: Primers used for sequence analysis of *IRF5*.

| **IRF5 Exon** | **Primer sequence** | **Amplicon size (bp)** | **PCR conditions** |
| --- | --- | --- | --- |
| Exon2a-F | 5’TGGACTGGAGAGACCATCCT3’ | 259 | **Step 1**: 1 cycle  10mins at 95°C  **Step 2**: x40 cycles  30sec at 95°C  25sec at 60°C  45sec at 72°C  **Step 3**: 1 cycle  5mins at 72°C |
| Exon2a-R | 5’CTCCAGGGGATGCAGAATAA3’ |
| Exon2b-F | 5’CAGGTGAACAGCTGCCAGT3’ | 206 |
| Exon2b-R | 5’TGCCTGTGTGCGTTATGTG3’ |
| Exon3a-F | 5’TATGGGACAGGAGGCAGACT3’ | 250 |
| Exon3a-R | 5’CCATTGGAGCAGACCTCGTA3’ |
| Exon3b-F | 5’CAAGTGGAAGGCCAACCTG3’ | 249 |
| Exon3b-R | 5’CTGGACCACTGCCTGCTAGA3’ |
| Exon4-F | 5’ATCTTGCTTCTCCTCCGACA3’ | 196 |
| Exon4-R | 5’GGAAACCTAAGGCCGATGG3’ |
| Exon5-F | 5’CCCCAGGTCAGTGGAATAAC3’ | 200 |
| Exon5-R | 5’ACCCTAGCCTCCCATCAGC3’ |
| Exon6a-F | 5’GTGCCTGGGAGGCAGTTC3’ | 288 |
| Exon6a-R | 5’CAGGCTCCAGGACCTCAGA3’ |
| Exon6b-F | 5’CCCCACATGACACCCTATTC3’ | 260 |
| Exon6b-R | 5’TCTGGCAGGAGCTGTTCG3’ |
| Exon6c-F | 5’GGCTTCAGGGAGCTTCTCTC3’ | 182 |
| Exon6c-R | 5’ACCCTCCTTGCCAATCCTAC3’ |
| Exon7a-F | 5’ACTCCCTTGGGTGGGAAA3’ | 272 |
| Exon7a-R | 5’GAAGCGCACTTGCTCCAG3’ |
| Exon7b-F | 5’CCCTCACCATCAGCAACC3’ | 254 |
| Exon7b-R | 5’GCTCCAGAACACCTTGCACT3’ |
| Exon7c-F | 5’AGCGCTTCTACACGAACCAG3’ | 284 |
| Exon7c-R | 5’AAATCAAGGTCCCTAGTCAGAGG3’ |
| Exon8-F | 5’TCCTCCTGGCTGCCTCTT3’ | 250 |
| Exon8-R | 5’GGCCTAGCCCCAGGATTC3’ |
| Exon9a-F | 5’CCTCATGCACAGCTGGATCT3’ | 274 |
| exon9a-R | 5’CAACACCAAGGCCTGCTC3’ |
| Exon9b-F | 5’ATGGTGGAGCAATTCAAGGA3’ | 210 |
| Exon9b-R | 5’GGGCTGTCACATCTCCACA3’ |

F: forward, R: Reverse.
