# Supplementary material for: An Integrated Genomic and Expression Analysis of 7q Deletion in Splenic Marginal Zone Lymphoma
Source: PLoS One. 2012 Sep 13;7(9):e44997. doi: 10.1371/journal.pone.0044997 (PMC3441634; doi:10.1371/journal.pone.0044997)
Supplement: Table S5 — Univariate analysis for prognosis by Kaplan-Meier method. (DOC) [file pone.0044997.s010.doc]

**Supplementary Table S5**: Univariate analysis for prognosis by Kaplan-Meier method.

|  | **Overall survival** | | **Progression free survival** | |
| --- | --- | --- | --- | --- |
|  | 5 year | *p* value | 5 year | *p* value |
| Sex | | | | |
| Male (n=18) | 0.86 | 0.78 | 0.78 | 0.96 |
| Female (n=48) | 0.71 |  | 0.62 |  |
| Age | | | | |
| <59 (n=18) | * | 0.026 | * | 0.04 |
| ≥60 (n=47) | 0.73 |  | 0.68 |  |
| Clinical Stage | | | | |
| <IV (n=4) | 0.67 | 0.4 | 0.67 | 0.96 |
| IV (n=51) | 0.82 |  | 0.77 |  |
| 7q deletion |  |  |  |  |
| Yes (n=26) | 0.79 | 0.45 | 0.78 | 0.78 |
| No (n=39) | 0.83 |  | 0.76 |  |

* No statistics are computed because all cases are censored (All death events occurred in patients with age ≥60). The analysis was performed using SPSS v 20.
